# Perinatal Exposure to Neuregulin-1 Results in Disinhibition of Adult Midbrain Dopaminergic Neurons: Implication in Schizophrenia Modeling

**DOI:** 10.1038/srep22606

**Published:** 2016-03-03

**Authors:** Hisaaki Namba, Takeshi Okubo, Hiroyuki Nawa

**Affiliations:** 1 Department of Molecular Neurobiology, Brain Research Institute, Niigata University, Niigata, 951-8585.

## Abstract

Aberrant neuregulin-1 (NRG1) signals are suggested to associate with the neuropathophysiology of schizophrenia. Employing a mouse schizophrenia model established by neonatal neuregulin-1 challenge, we analysed postpubertal consequence of the NRG1 pretreatment for the electrophysiological property of nigral dopamine neurons. *In vivo* single unit recordings from anaesthetized NRG1-pretreated mice revealed increased spike bursting of nigral dopamine neurons. In slice preparations from NRG1-pretreated mice, spontaneous firing was elevated relative to controls. The relative increase in firing rates was abolished by a GABA_A_ receptor antagonist. Whole-cell recording showed that perinatal NRG1 pretreatment diminished inhibitory miniature synaptic currents as well as GABA_A_ receptor sensitivity. These results collectively suggest that perinatal exposure to neuregulin-1 results in the disinhibition of nigral dopamine neurons to influence their firing properties at the adult stage when the behavioral deficits are evident.

Neuregulin-1 (NRG1) is one of candidate genes for schizophrenia susceptibility^1^. Genetic linkage studies have demonstrated an association with schizophrenia, although there are conflicting reports among ethnic populations^2^. Neuropathologic and clinical examinations revealed aberrant NRG1 signalling in patients with schizophrenia^3^,^4^. Despite the accumulated pathologic and genetic evidence for the association between NRG1 dysregulation and schizophrenia, the neurobiological underpinnings of this association remain largely uncharacterized^5^.

In the central nervous system, a NRG1 receptor, ErbB4, is highly expressed in neocortical GABA neurons and midbrain dopaminergic neurons, whereas another NRG1 receptor, ErbB3, is mainly expressed in glial cells such as oligodendrocytes^6^,^7^,^8^,^9^. Biochemical and physiological influences of NRG1 have been intensively investigated in GABAergic cell populations, and such studies have implicated dysregulation of GABAergic transmission in the pathophysiology of schizophrenia, supporting the ‘GABA hypothesis’. The impact of endogenous NRG1 is most evident on parvalbumin-positive GABA neurons in the neocortex, where NRG1 signals enhance inhibitory synaptic transmission and GABAergic development^10^,^11^,^12^,^13^,^14^. In contrast to the knowledge on the NRG1 actions in GABAergic neurons, the information of NRG1 signals in midbrain dopaminergic neurons is limited^13^,^14^.

According to the neurodevelopmental hypothesis for schizophrenia, we established a mouse schizophrenia model by treating neonatal mice with NRG1. Following peripheral administration, a mature form of NRG1 or an epidermal growth factor (EGF)-like core domain of NRG1 (eNRG1) penetrates the blood–brain barrier of the neonates and activates ErbB4 in the midbrain and neocortex^14^,^15^,^16^,^17^. The mice that are perinatally exposed to eNRG1 later exhibit behavioural deficits relevant to schizophrenia at the postpubertal stage, including decreased prepulse inhibition and latent inhibition of fear learning as well as abnormal social interactions^14^. We found that this animal model also exhibits abnormalities in dopamine innervation as well as in hypersensitivity to psychostimulants^14^,^16^. However, how these phenotypic changes involve pathophysiological processes of dopaminergic neurons are not yet determined.

In the present study, we characterized the long-term effects of perinatal eNRG1 pretreatment on the electrophysiological properties of adult nigral dopamine neurons by *in vivo* extracellular single unit recordings (in the anaesthetized state) and by *in vitro* single unit recordings and whole-cell recordings in midbrain slices. To distinguish the intrinsic effects from the extrinsic effects of synaptic inputs, we measured miniature synaptic currents in this cell population. These electrophysiological results implicate NRG1-mediated alterations in dopaminergic activities in the schizophrenia-associated behaviours of this model. We also discuss provisional similarities of the present dopaminergic pathophysiology to the mechanism of opioid addiction.

## Results

### Perinatal eNRG1 challenge results in spike burst elevation of nigral dopamine neurons

**Figure 1:**
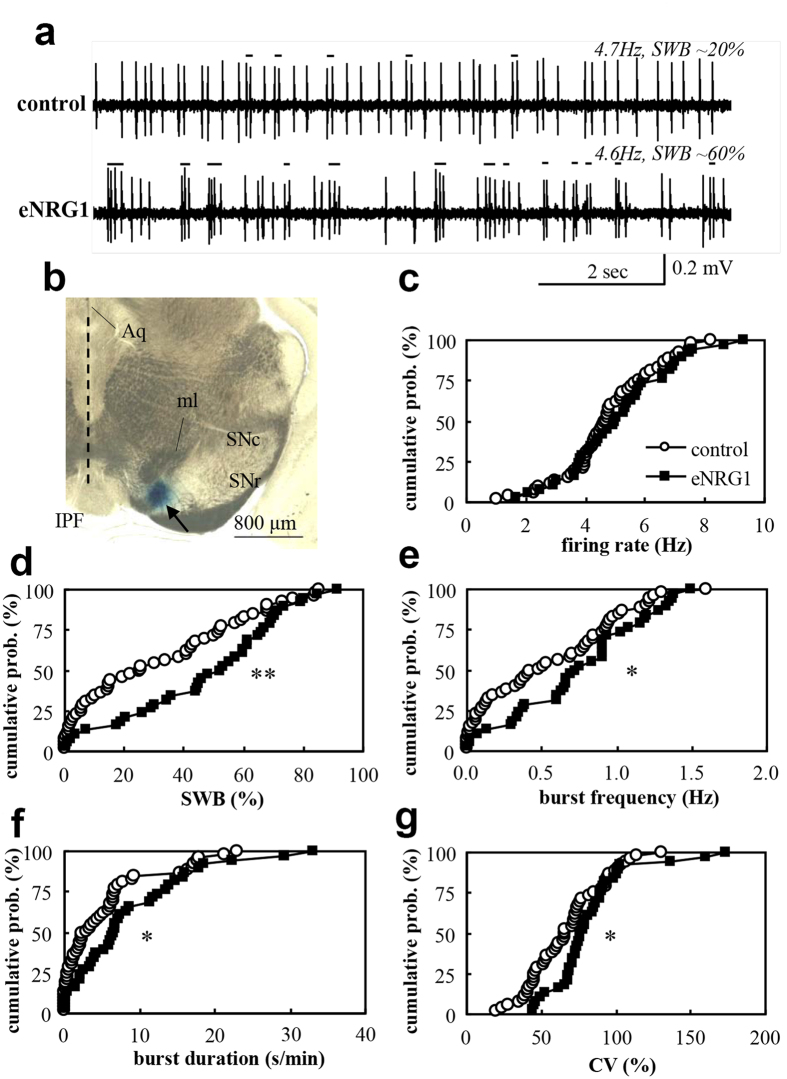
Perinatal eNRG1 administration results in higher burst activity of adult nigral dopamine neurons *in vivo*. (**a**) Typical single unit traces with similar firing rates are displayed for control and eNRG1-pretreated mice. The bursting periods are marked with black lines. (**b**) A recording site marked with Pontamine sky blue. Aq: aqueduct, IPF: interpeduncular fossa, ml: medial lemniscus, SNr: substantial nigra pars reticulata. Mean firing rates (**c**), spikes within bursts (SWB) (**d**), burst frequency (**e**), burst duration (**f**) and coefficient of variation (CV) of interspike intervals (**g**) are plotted for controls and eNRG1-pretreated mice. Dopaminergic neurons from eNRG1-pretreated mice exhibited a greater proportion of total spikes within bursts (**d**) as well as more bursts, whereas mean firing rates did not differ from dopaminergic neurons in control mice (n = 38 cells from 7 control mice and n = 53 cells from 11 eNRG1-pretreated mice). *p < 0.05, **p < 0.01, Mann–Whitney U test.

We subcutaneously administered recombinant eNRG1 protein to mouse pups as described previously^14^,^15^,^16^. Mice were grown to young adults when these mice are reported to exhibit the behavioural deficits relevant to schizophrenia endophenotypes^14^,^16^. We performed *in vivo* single unit recordings from anaesthetized adult controls and eNRG1-pretreated mice (Fig. 1). Nigral dopamine neurons exhibited spontaneous activity with the mean firing rate of 4.8 ± 0.2 Hz and a burst ratio of 31 ± 4% in control mice and with a mean firing rate of 5.1 ± 0.3 Hz and a burst ratio of 67 ± 4% in eNRG1-pretreated mice. Although there was no significant difference in mean firing rates between groups (Fig. 1c), these neurons in eNRG1-pretreated mice showed a higher percentage of spikes within bursts (SWB) (Fig. 1d), greater frequency of bursts (Fig. 1e), longer burst duration (Fig. 1f), and larger coefficient of variation (CV) of interspike intervals (Fig. 1g). Thus, perinatal eNRG1 signals appear to shift the spontaneous spiking behaviour from tonic to bursting mode.

### Firing of dopamine neurons is enhanced in midbrain slices from eNRG1-pretreated mice

**Figure 2:**
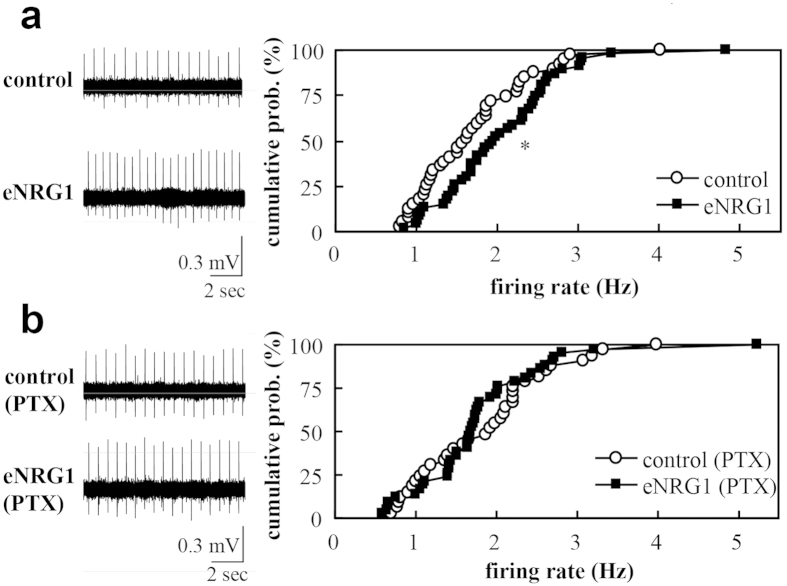
Effects of perinatal eNRG1 administration on firing rates of dopaminergic neurons in midbrain slices. Mean firing rates of single units are compared in the presence (**b**) or absence (**a**) of 50 μM picrotoxin (PTX). Single units were recorded *in vitro* in the anterior region of medial terminal nucleus of the accessory optic tract. Typical single unit traces are displayed (left panels in **a**, **b**). (**a**) Cumulative probability distributions of firing rates are compared between control and eNRG1 groups in the control condition (n = 39 cells from 4 control mice and n = 42 cells from 5 eNRG1-pretreated mice). (**b**) Cumulative probability distributions of firing rates in the presence of PTX are compared (n = 33 cells from 4 control mice, n = 46 cells from 5 eNRG1-pretreated mice). *p < 0.05, Mann–Whitney U tests. Note; Coefficient variation of interspike intervals was 6.2 ± 0.6% for control, 4.5 ± 0.5% for eNRG1, 4.5 ± 0.5% for control plus PTX, and 4.7 ± 0.5% for eNRG1 plus PTX (F_3,157_ = 3.2, p = 0.023, ANOVA, post-hoc; p < 0.05 for a PTX effect in control group).

The shift in firing mode (an increase in bursting activity) may result from changes in intrinsic firing properties and/or changes in synaptic inputs onto dopaminergic neurons. To distinguish these possibilities, we performed single unit recordings in midbrain slice preparations. Dopamine neurons in slices from eNRG1-pretreated mice exhibited a significantly higher mean spike frequency than dopamine neurons in control slices (2.1 ± 0.1 Hz vs 1.7 ± 0.1 Hz, p < 0.05, Mann–Whitney U-test; Fig. 2a). The firing rates *in vitro* were markedly reduced, compared with those *in vivo* (p < 0.001, Mann–Whitney U-test). No spike bursting was observed in slice conditions, consistent with previous studies^18^. Bath perfusion of a GABA_A_ receptor antagonist, picrotoxin (PTX, 50 μM), marginally reduced mean spike frequency in eNRG1 group (1.8 ± 0.1 Hz, p = 0.055, Mann–Whitney U-test) but not in control group (1.9 ± 0.1 Hz, p = 0.5, Mann–Whitney U-test). Accordingly, PTX perfusion abolished the frequency difference between eNRG1 and control groups (Fig. 2b). These results suggest that the higher firing activity in eNRG1-pretreated mice might involve the attenuation of synaptic GABAergic inhibition (i.e. disinhibition).

**Figure 3:**
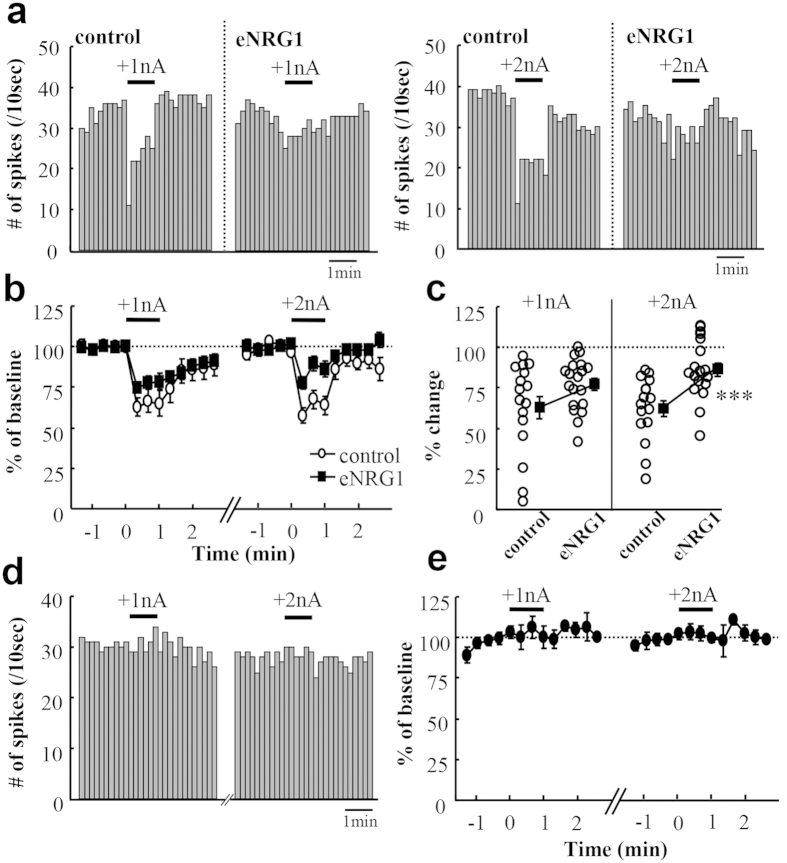
eNRG1 effects on GABA_A_ receptor agonist sensitivity of nigral dopamine neurons *in vivo*. We examined the sensitivity of *in vivo* dopaminergic firing to muscimol under anaesthetic condition. (**a**) Typical effects of iontophoretic application of muscimol on the spontaneous firing activity are displayed in control and eNRG1-pretreated mice. The drug was applied for 1 min (horizontal bars) to the same cell with the driving current of +1 nA (left panel) and then +2 nA (right panel). (**b**) Firing rates were averaged for every 20 sec before and after local muscimol application. Mean firing rates for 1 min before drug application were set to 100% for each cell (n = 16 cells from 6 control mice and n = 20 cells from 6 eNRG1-pretreated mice). (**c**) The responses to muscimol (+1 nA and +2 nA) were averaged during the period of the 1 min current ejection and plotted. (**d**) We performed iontophoretic application of vehicle alone to a control mouse (1 mM NaCl, pH 4.0) with the driving current of +1 nA (left panel) or +2 nA (right panel). Histograms of typical spike rates are displayed. (**e**) Effects of control iontophoresis are averaged and shown (n = 5 cells from one mouse). *p < 0.05, **p < 0.01, ***p < 0.001, Mann-Whitney U test.

To confirm the above hypothesis raised by the *in vitro* results, we explored *in vivo* effects of a GABA_A_ receptor agonist on nigral dopamine neurons (Fig. 3). By iontophoresis with a parallel glass electrode, we locally infused muscimol into the local area where a unit recording electrode was located. The microinfusion of muscimol with 2 nA currents, but not with 1 nA currents, more markedly reduced the firing rates of nigral dopamine neurons in control mice than those in eNRG1-pretreated mice; the averaged changes in frequency were −37.7 ± 4.9% for control mice and −15.0 ± 4.3% for eNRG1-pretreated mice (p < 0.001, Mann–Whitney U-test). In contrast, the infusion of vehicle with either driver currents (1 nA or 2 nA) exhibited no significant effects on baseline frequency in control mice (Fig. 3d,e). These results suggest that dopamine neurons of eNRG1-pretreated mice are less susceptible to the GABA_A_ receptor agonist than those of control mice.

### Perinatal eNRG1 treatment diminishes GABA sensitivity of nigral dopamine neurons

**Figure 4:**
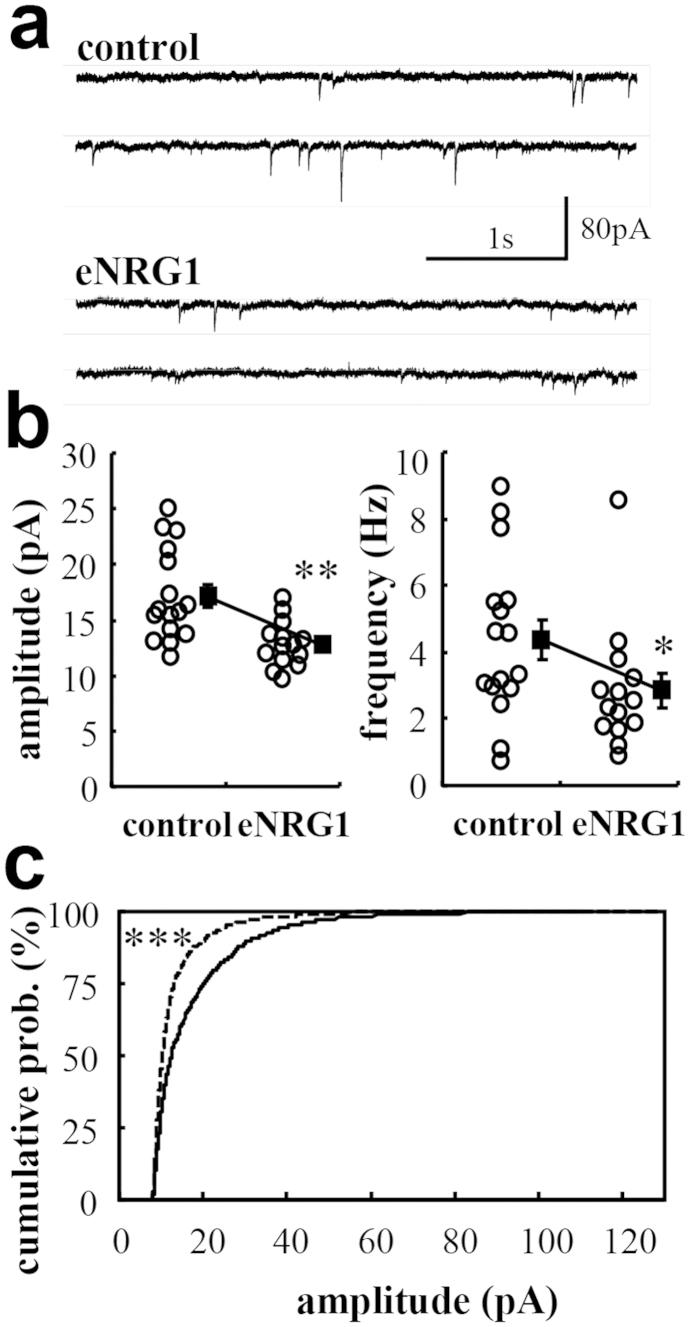
Effects of perinatal eNRG1 administration on miniature inhibitory synaptic currents in adult dopaminergic neurons. (**a**) Typical current traces are displayed as examples. (**b**) The amplitude and frequency of inhibitory miniature events are recorded from dopaminergic neurons and plotted (n = 16 cells from 6 control mice and n = 14 cells from 6 eNRG1-pretreated mice). (**c**) One hundred events were extracted from all individual cells and pooled. Cumulative probability distributions of their amplitudes are calculated for each group. *p < 0.05, **p < 0.01, Mann–Whitney U test, ***p < 0.0001 by Kolmogorov–Smirnov test.

To evaluate the inhibitory influences on dopaminergic firing, we measured miniature inhibitory postsynaptic currents (mIPSCs) (Fig. 4). The amplitude and frequency of mIPSCs were significantly diminished in slices from eNRG1-pretreated mice (p < 0.01 for amplitude, p < 0.05 for frequency, Mann–Whitney U-test). The decay time of averaged mIPSCs was significantly larger in eNRG1-pretreated mice (p < 0.01, Mann–Whitney U-test; Table 1).

**Figure 5:**
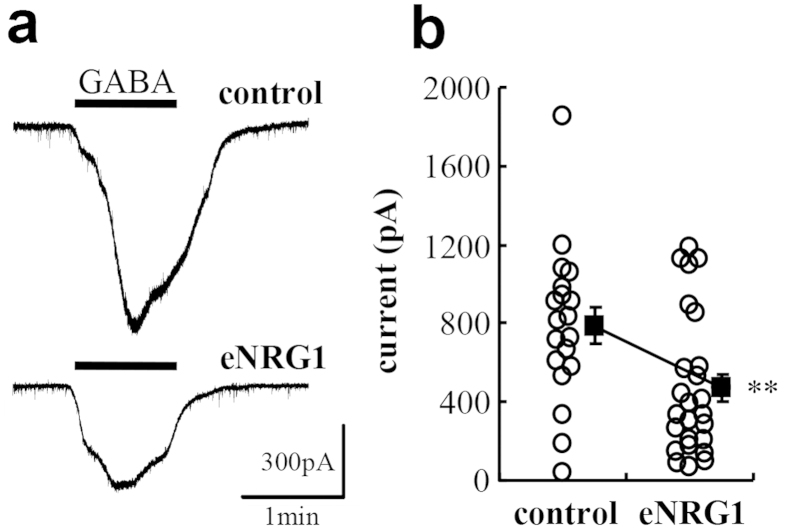
Reduced GABA_A_ receptor agonist sensitivity of nigral dopamine neurons from eNRG1-pretreated mice. (**a**) Inward currents were triggered by application of 1 mM GABA to midbrain slice preparations in the presence of the GABA_B_ receptor antagonist CGP52432. Typical traces are displayed. (**b**) The peak amplitudes are plotted and compared (n = 17 cells from 6 control mice and n = 23 cells from 8 eNRG1-pretreated mice). **p < 0.01 by Mann–Whitney U test. Note; in the presence of CGP52432, input resistance of eNRG1-pretreated cells was rather larger than control (control: 252 ± 18 MΩ; eNRG1: 391 ± 32 MΩ, p < 0.01), but no differences were detected in other electrophysiological properties (data not shown). The GABA_A_ receptor blocker PTX markedly reduced the GABA-evoked currents (see Supplemental Information Fig. S1).

To explore the mechanism(s) underlying these decreases in inhibitory synaptic events, we measured GABA-induced inward currents in dopaminergic neurons of midbrain slices (Fig. 5). Superfusion of 1 mM GABA to midbrain slices triggered significantly larger inward currents in control mice than eNRG1-pretreated mice (825 ± 97 pA vs 491 ± 76 pA; p < 0.05, Mann–Whitney U-test). The GABA-triggered inward currents were significantly blocked by the co-application of PTX (reduced to 10 ± 2%, p < 0.05, Mann–Whitney U-test)(Supplementary Information Fig. S1). These results suggest that the greater burst firing of dopaminergic neurons in eNRG1-pretreated mice presumably involves a decrease in the sensitivity of postsynaptic GABA_A_ receptors.

### Limited effects of eNRG1 on intrinsic membrane properties and NMDA receptor sensitivity of nigral dopamine neurons

**Figure 6:**
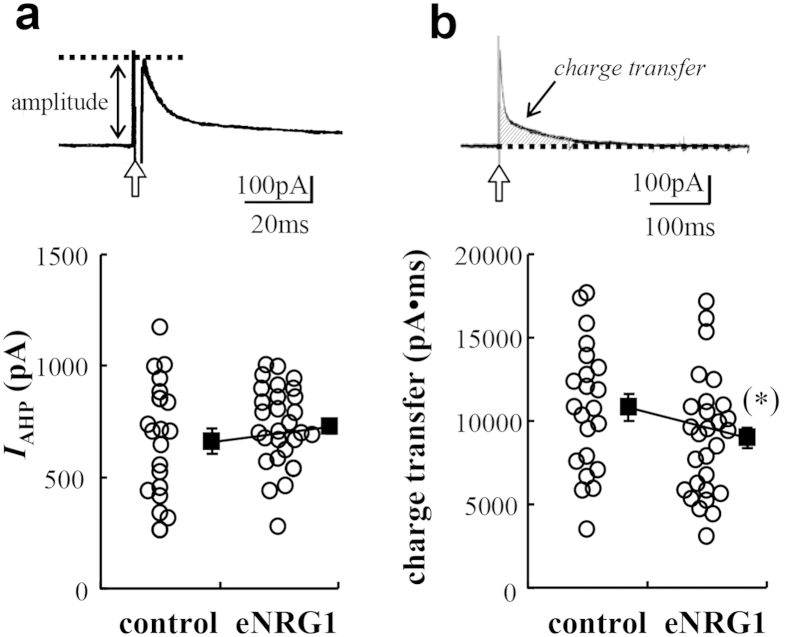
Effects of perinatal eNRG1 pretreatment on *I*_AHP_ in adult dopaminergic neurons. (**a,b**) Unclamped action potentials were elicited in nigral dopamine neurons of control and eNRG1-pretreated mice. Depolarizing pulses (duration 2 ms, marked with arrows) from a holding potential of −55 mV to +40 mV were given. The peak amplitude (**a**) and total charge transfer (**b**) of the outward tail current were measured (n = 22 cells from 9 control mice, n = 30 cells from 12 eNRG1-pretreated mice). The peak amplitude was not affected (662 ± 56 pA for control, 727 ± 33 pA for eNRG1, p = 0.30, Mann-Whitney U test), but the total charge transfer was marginally decreased in eNRG1-pretreated mice (10773 ± 816 pA•ms for control, 8978 ± 647 pA•ms for eNRG1, (*) p = 0.066, Mann-Whitney U test).

We examined if changes in intrinsic membrane properties might also contribute to the enhanced burst firing of nigral dopamine neurons in anaesthetized eNRG1-pretreated mice. We observed no significant difference in series resistance (Rs), membrane resistance (Rm) or membrane capacitance (Cm) between control and eNRG1-pretreated mice (Table 1). We also recorded a hyperpolarizing activated current, *I*_h_ (Table 1), and an after-hyperpolarizing current (*I*_AHP_) (Fig. 6), both of which regulate firing frequency and the temporal fidelity of pacemaker-like activity in these neurons^19^,^20^ and are cardinal electrophysiological features of dopaminergic neurons in substantia nigra^20^. No significant differences were detected in either *I*_h_ or *I*_AHP_ between control and eNRG1-pretreated groups.

**Figure 7:**
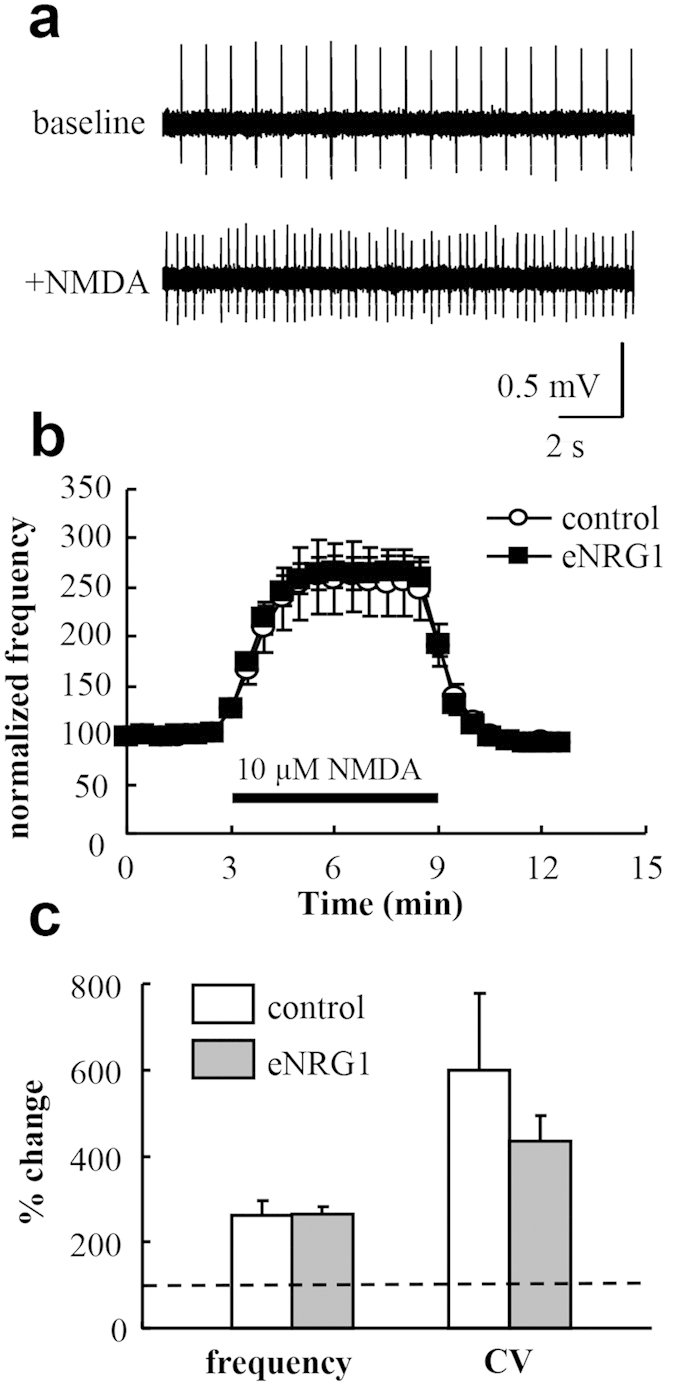
Effects of perinatal eNRG1 pretreatment on NMDA receptor sensitivity in dopaminergic neurons. Midbrain slices were prepared from control and eNRG1-pretreated mice and single unit activities were monitored from dopaminergic neurons in the presence or absence of 10 μM NMDA. (**a**) Typical single unit traces are displayed. (**b**) The time dependency of the NMDA effects is displayed. Mean firing rates before NMDA application were set to 100% in each cell (n = 13 cells from 4 control mice, n = 17 cells from 4 eNRG1 pretreated mice). (**c**) Mean frequency and coefficient of variation (CV) of interspike intervals before NMDA application were set to 100% (a horizontal broken line) and compared with those after NMDA application (from 4 min to 6 min) (p = 0.47 for frequency; p = 0.71 for coefficient variation, Mann–Whitney U test).

To assess the contribution of excitatory neurotransmission to the irregularity of dopaminergic firing *in vivo*^21^, we also examined the influences of NMDA on firing properties of dopaminergic neurons in midbrain slice preparations (Fig. 7). Superfusion of 10 μM NMDA similarly elevated the frequency and irregularity of dopaminergic firing in both control and eNRG1-pretreated mice but eNRG1 effects were not detected (p = 0.47 for frequency, p = 0.71 for coefficient of variation, Mann–Whitney U-test) (Fig. 7b,c). These results suggest that the effects of neonatal eNRG1-pretreatment on NMDA receptors were limited in nigral dopamine neurons. Thus, perinatal eNRG1 challenge had no apparent effects on intrinsic membrane excitability and postsynaptic NMDA receptor sensitivity of nigral dopamine neurons in the present model.

## Discussion

Our previous reports demonstrated that NRG1 penetrates the immature blood–brain barrier following subcutaneous administration to mouse pups, directly acts on ErbB4 receptors in the midbrain and promotes terminal arborization of midbrain dopaminergic neurons^7^,^8^,^14^. These changes are temporally correlated with the abnormal behaviours that emerge in early adulthood. The present experiments revealed that neonatal exposure to eNRG1 results in electrophysiological changes in the firing properties of nigral dopamine neurons. The present results of eNRG1-pretreated mice include (1) increased SWB relative to total spikes and higher frequency of bursts *in vivo*; (2) higher spike frequency *in vitro* which was abolished by pharmacological blockade of the GABA_A_ receptor; (3) less remarkable impact of *in vivo* microinfusion of a GABA_A_ receptor agonist; (4) no changes in membrane characteristics (*I*_*h*_ currents, *I*_*AHP*_ currents, or membrane resistance); (5) decreases in mIPSC amplitudes and frequencies, (6) a substantial decline of GABA_A_ receptor agonist-evoked currents, and (7) no significant differences in NMDA effects in slice. These results suggest that perinatal NRG1 treatment results in the reduction in synaptic GABA neurotransmission, which presumably involves the decreased channel activity of GABA_A_ receptors in these neurons and may contribute to the elevated burst firing activity of dopamine neurons *in vivo*. The *in vivo* and *in vitro* effects of eNRG1 on firing properties of dopaminergic neurons were both stimulatory for their function but qualitatively differed. Thus, this controversy remains to be characterized further.

Given the enrichment of the ErbB4 receptor in midbrain dopaminergic neurons, we postulate that the observed intrinsic effects on GABA receptor sensitivity mainly represent the direct actions of NRG1 on nigral dopamine neurons, whereas the *in vivo* effects on dopaminergic bursting may include extrinsic influences as well (see below). As we failed to find any significant influence of eNRG1 on *I*_h_ or *I*_AHP_ , as well as on NMDA-receptor sensitivity, the intrinsic influences of eNRG1 appear to be limited to GABA_A_ receptor function. These pharmacological results from the GABA_A_ receptor agonist and antagonist agree with the decreases in mIPSC amplitudes, although the decreases in mIPSC frequency may implicate the additional extrinsic influence of inhibitory afferents.

Burst firing was observed many weeks after eNRG1 exposure, while acute eNRG1 application had no significant effects on the spiking properties of dopaminergic neurons (Supplemental Information Fig. S2). Why did the dopaminergic hyposensitivity to GABA emerge with such a time delay? Currently, we do not have a definitive answer. However, Okada *et al.*^22^ reported a similar NRG1 effect on GABA_A_ receptors^22^. Stimulation of postnatal hippocampal neurons with NRG1 attenuated the expression of GABA_A_ receptor mRNAs. This down-regulation appeared to require prolonged treatment with NRG1^22^. Accordingly, we speculate that the hyposensitivity of eNRG1 pretreated mice to GABA reflects an irreversible overgrowth of developing dopaminergic neurons in response to subchronic NRG1 stimulation. In agreement with this hypothesis, our initial assessment of this model mouse indicated excess arborization of the dopaminergic fibres during development. However, we do not rule out the indirect influences of eNRG1 on inhibitory and excitatory afferents innervating the substantia nigra from a distance with the given wide distributions of ErbB4^23^,^24^.

Firing patterns and excitability of *in vivo* and *in vitro* dopaminergic neurons are regulated by intrinsic channel properties as well as by extrinsic synaptic inputs^25^. The frequency and regularity of spiking are controlled not only by the hyperpolarization-activated current *I*_h_ and GABAergic synaptic inputs but also by NMDA receptors and several potassium channels^19^,^21^,^26^,^27^,^28^,^29^,^30^,^31^,^32^. With the given slice condition, both excitatory and inhibitory afferents from the striatum and neocortex were removed prior to *in vitro* recording. The denervation of major excitatory inputs to nigral dopamine neurons may illustrate the observed controversy that the eNRG1 effects *in vitro* appeared in firing rates but not in burst rates^24^,^25^. In agreement with this controversy, our recording from midbrain slice preparation exhibited a marked reduction in firing frequency and irregularity of nigral dopamine neurons, compared with the relevant *in vivo* data. In this context, the impact of the local disinhibition on nigral dopamine neurons remains to be compared with that of GABAergic afferents distantly projecting to these cells.

A similar phenomenon was reported in response to local infusion of another neurotrophic factor, glial derived neurotrophic factor (GDNF), for which specific receptors are also enriched in midbrain dopaminergic neurons. The GDNF-induced enhancement of dopaminergic firing results from the alteration of GABAergic afferents as well as from postsynaptic GABA sensitivity^28^. A reduction in postsynaptic GABA sensitivity may be the general consequence of neurotrophic responses of this cell population^28^,^29^. Enhanced burst activity of dopaminergic neurons has also been reported in other rodent models of schizophrenia^33^,^34^. Thus, dysregulation of the dopamine system appears to be a common pathomechanism underlying behavioural endophenotypes of schizophrenia^35^,^36^, although this ‘dopamine hypothesis’ has often been challenged in favour of ‘glutamatergic’ and ‘GABA’ hypotheses^37^,^38^. Elevated spike bursting of dopaminergic neurons presumably results in higher dopamine release and elevates the propensity for aberrant behaviours^39^. However, single unit measurements in freely moving (unanesthetized) mice are required to precisely assess the contribution of dopaminergic burst firing to neurobehavioral dysfunction^40^.

Disinhibition of midbrain dopaminergic neurons is also strongly implicated in the pathophysiology of opioid addiction^41^. Opioid receptor ligands are often hallucinogenic, inducing perceptual distortions, depersonalization and speech/language impairments in human^42^,^43^. In rodents, these agonists cause disrupted PPI of the acoustic startle as well as aggression and abnormal social behaviours^44^,^45^,^46^, which are found in the present eNRG1-pretreated mice^14^,^15^,^16^. Some of these opioid-induced cognitive and behavioural abnormalities have been ascribed to hyper-dopaminergic activity driven by disinhibition of GABAergic afferents^47^,^48^. It is noteworthy that the opioid-induced and NRG1-induced schizophrenia-like behaviours both involve disinhibition of GABA inputs to midbrain dopaminergic neurons. This shared mechanism may further strengthen the validity of this NRG model as a tool to investigate the pathophysiology of schizophrenia.

## Materials and Methods

### Subjects

Pregnant C57Bl/6NCr mice (at gestation day 15 or 16) were purchased from SLC (Shizuoka, Japan). Experimental pups were administered daily subcutaneous (s.c.) injections of the human recombinant EGF-like core domain of NRGβ1 (eNRG1; 1.0 mg/kg, PeproTech, London, UK) from postnatal (P) days 2–10^16^. Control pups received daily injections of vehicle. Mice were grown in a temperature-controlled colony room (22.0 ± 1.0 °C) and maintained under a 12-h light-dark cycle (8:00 on −20:00 off). All of the animal experiments described here were approved by the Animal Use and Care Committee of Niigata University and performed in accordance with the Guiding Principles for the Care and Use of Laboratory Animals (NIH, USA). All efforts were made to minimize both the suffering and number of animals used in this study.

### *In vivo* recording

Male mice (12–14 weeks old, eNRG1- or vehicle-pretreated) were anaesthetized with an intraperitoneal (i.p.) injection of trichloroacetaldehyde monohydrate (chloral hydrate, 400 mg/kg, Wako, Osaka, Japan) and placed in a stereotaxic apparatus (SR-6M, Narishige) with an auxiliary ear bar for mice (EB-5N, Narishige, Tokyo, Japan). Body temperature was maintained at 36–37 °C using a heating pad. The skull overlying the midbrain was removed [Anterior–Posterior (AP) −2.4 to −3.8 mm from bregma; Medial–Lateral (ML) 0.2 to 1.6 mm from bregma). Extracellular action potentials were recorded with glass microelectrodes filled with 2% Direct Blue 1 (Pontamine Sky Blue 6B; Tokyo Kasei, Tokyo, Japan) in 0.5 M NaCl. Electrode tips were broken to adjust the resistance to 10–18 MΩ. Extracellular potentials originating from putative dopaminergic neurons were identified by well-established criteria^49^,^50^. Recording and analysis procedures for comparing spontaneous activity between dopaminergic neurons from eNRG1- and vehicle-pretreated mice were modified from Mameli-Engvall *et al.*^50^. The electrode was passed repeatedly (6 times per mouse) at varying stereotaxic AP-ML coordinates (AP: −2.7 to −3.0 mm from bregma, ML: 0.8 to 1.0 mm from bregma) separated by 100 μm to reach 4.2–5.0 mm below the right cortical surface.

Each dopaminergic neuron was recorded for 2 min after a 2-min stabilization period. After recording from each track, Pontamine Sky Blue dye was iontophoretically injected (−20 μA for 15 min). After cardiac perfusion of 10% formalin (Wako) in 0.1 M phosphate buffer, the brain was removed and fixed with the same solution. To determine the recording position, midbrain slices (100-μm thickness) were prepared and bright field images of dye deposits were photographed using a digital microscope with reconstruction mode (Keyence BZ-9000, Osaka, Japan). Stereotaxically determined ML levels were corrected by measuring the distance from the dye-injection site to the midline. We analysed cells located at ML 0.8–1.0 mm right, identified as the medial SNc and anterior parabrachial pigmented nucleus (see Fig. 1b)^51^.

Spike units originated from dopaminergic neurons were identified by well established criteria, i.e., their rather longer duration; ≥1.1 msec from an onset to a negative peak and occasional bursting activities with lower firing rates^36^. Spike units with shorter duration (<1.1 ms) were excluded in the present analysis. As an index of bursting, we counted the proportion of spikes occurring within bursts (‘spikes within bursts’, SWB). The period of the firing bursts was identified with the following criteria; (1) The onsets are defined by two consecutive spikes within an interval lower than 80 msec and whenever (2) they terminated with an interval greater than 160 msec^49^. The number of spikes in this bursting period was counted as spikes within bursts (SWB).

### Drug application *in vivo*

A double-barrelled theta glass microelectrode (TGC-150, Harvard Apparatus, Edenbridge, UK) was employed for iontophoretic application to a recording site. One of the barrel for drug application was filled with 10 mM muscimol (Wako, dissolved with 0.1 M NaCl, pH4.0)^52^ and the other barrel for recording contained 0.5M NaCl containing with 2% Pontamine Skyblue Dye. The electrode (15–25 MΩ) was lowered into substantia nigra as described above. After baseline recordings for 2 min, muscimol was delivered by +1 nA and +2 nA currents for 1 min to the same cells. At the distinct recording position, the second drug application was done at least 5 min after the first drug application. A retaining current of −5nA was constantly given between muscimol ejections.

### Single unit recording from midbrain slices

Adult mice (10–15 weeks old, eNRG1- or vehicle-pretreated) were anaesthetized with halothane and decapitated. Horizontal midbrain slices (thickness: 400 μm) at the level of the interpeduncular fossa were prepared as described^53^,^54^. The slices were exposed to 34 °C Krebs solution for 30 min and then maintained at room temperature (24–26 °C). Immediately before recording, slices were placed in a chamber continuously perfused at ~3 ml/min with 32 ± 1 °C normal Krebs solution.

Single unit recordings were obtained from the anterior region of the medial terminal nucleus of the accessory optic tract (MT), which contains dopaminergic cell bodies of the substantia nigra pars compacta^55^. Glass microelectrodes were filled with 2 M NaCl. A glass microelectrode (10–20 MΩ) was placed in the anterior region of MT and manipulated into the slice using a water hydraulic micromanipulator (WR-6, Narishige). Spike units originating from putative dopaminergic neurons were identified as described above^50^. Spikes <2 ms in duration were excluded from the analysis. To evaluate the contribution of GABA transmission, measurements were performed in the presence and absence of 50 μM PTX (Sigma, St Louis, MO, USA). Spike events were detected using Mini Analysis Program (Jaejin Software, Leonia, NJ). As an index of the regularity of spike firing, coefficient of variation of inter-spike intervals was calculated by dividing the standard deviation of the Gaussian fit by the mean inter-spike interval (expressed as %)^56^. Dopaminergic neurons were identified as described above.

### Whole-cell patch-clamp recording

One or two midbrain horizontal slices (300-μm thick) were prepared from each mouse (10–15 weeks olds). Whole-cell patch-clamp recordings were made at room temperature from putative dopaminergic neurons located in the medial or anterior region of MT^51^. Dopaminergic neurons were identified by a prominent *I*_h_ activated by hyperpolarizing pulses (70 mV, duration: 800 ms) from a holding potential of −55 mV as well as by *post hoc* immunohistochemical analysis of biocytin labelled cells (see below).

mIPSCs were recorded at a holding potential of −60 mV using patch pipettes filled with a high chloride internal solution modified from Mansvelder *et al.*^57^ (65 mM potassium methansulfonate, 65 mM KCl, 10 mM HEPES, 0.2 mM EGTA, 4 mM Mg-ATP and 5 mM biocytin-Cl, 280–300 mOsm, pH 7.4). The external solution contained 50 μM DL-2-amino-5-phosphonopentanoic acid (DL-AP5; Tocris Bioscience, Bristol, UK), 10 μM 6-cyano-7-nitroquinoxaline dione (CNQX; Tocris Bioscience), and 1 μM tetrodotoxin (TTX; Wako). Synaptic events were detected with a threshold level of 8 pA by the Mini Analysis Program (Synaptosoft, Fort Lee, NJ, USA). To determine mIPSC kinetics for each cell, more than 50 miniature events were selected to reject double peaks and averaged. Amplitudes of miniature responses were determined from baseline to peak. The time constants of single exponential fits were used to describe the decay time. The rise time was estimated as the time necessary to rise between 10 and 90% of the peak response.

GABA currents evoked by superfusion of 1 mM GABA (Wako) were recorded at a holding potential of −55 mV in the presence of the GABA_B_ receptor antagonist CGP 52432 (10 μM, Abcam, Cambridge, UK). After recording, slices were fixed and processed for immunostaining with anti-tyrosine hydroxylase (TH) monoclonal antibody to confirm somatic positions and the TH-immunoreactivity (dopaminergic phenotype) of biocytin-labelled cells.

To elicit and measure *I*_AHP_, unclamped action potentials were evoked by depolarizing pulses (duration; 2 ms) from the holding potential to +40 mV^58^. The amplitude and charge transfer of the outward tail current were measured to estimate the fast and slow after-hyperpolarization components, respectively^58^.

### Statistics

Results are presented as mean ± S.E.M. Electrophysiological data from eNRG1-pretreated and vehicle-pretreated mice were compared by Mann–Whitney U tests and/or Kolmogorov–Smirnov tests. P < 0.05 was considered statistically significant. Group effects of coefficient variation values were estimated with an analysis of variance (ANOVA). Statistical analysis was performed using SPSS software (ver. 11.5; SPSS Japan Inc., Tokyo, Japan).

## Additional Information

**How to cite this article**: Namba, H. *et al.* Perinatal Exposure to Neuregulin-1 Results in Disinhibition of Adult Midbrain Dopaminergic Neurons: Implication in Schizophrenia Modeling. *Sci. Rep.* **6**, 22606; doi: 10.1038/srep22606 (2016).

## Supplementary Material

Supplementary Information

## Figures and Tables

**Table 1:** Electrophysiological properties and hyperpolarizing activated currents of nigral dopamine neurons in midbrain slices.

	Rs (MOhm)	Rm (MOhm)	Cm (pF)	I_*h*_ (pA)	kinetics of mIPSCs	
					rise (ms)	decay (ms)
control(n = 16)	14.8 ± 0.7	366 ± 37	122 ± 6	425 ± 60	1.4 ± 0.1	6.9 ± 0.5
eNRG1(n = 14)	14.7 ± 0.7	311 ± 53	108 ± 9	437 ± 74	1.9 ± 0.2(*)	8.5 ± 0.5**

Membrane potential was clamped at a holding potential of −60 mV in the presence of 1 μM tetrodotoxin and glutamate receptor blockers. Rs: series resistance; Rm: membrane resistance; Cm: membrane capacitance. Rise and decay times of mIPSCs were measured from averaged miniature currents in each cell. Mean ± SEM. (*): p = 0.063; **p < 0.01 by Mann-Whitney U test.
