# Supplementary material for: Perinatal Exposure to Neuregulin-1 Results in Disinhibition of Adult Midbrain Dopaminergic Neurons: Implication in Schizophrenia Modeling
Source: Sci Rep. 2016 Mar 3;6:22606. doi: 10.1038/srep22606 (PMC4776181; doi:10.1038/srep22606)
Supplement: Supplementary Information [file srep22606-s1.pdf]

Supplemental Information

Perinatal Exposure to Neuregulin-1 Results in Disinhibition of Adult Midbrain Dopaminergic Neurons: Implication in Schizophrenia Modeling

Hisaaki Namba, Takeshi Okubo, Hiroyuki Nawa

Supple Fig S1; Picrotoxin (PTX) sensitivity of GABA-triggered inward currents in the presence of 10 µM CGP52432.

Supple Fig S2; Acute eNRG1 application had no marked influence on single unit activity of nigral dopamine neurons.

Suppl. Fig. S1

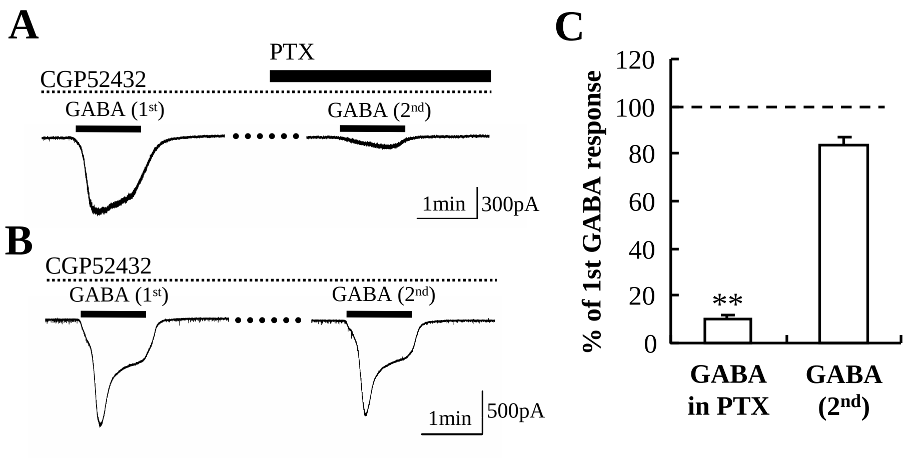

Suppl. Fig. S1
Picrotoxin (PTX) sensitivity of GABA-triggered inward currents in the presence of 10 µM CGP52432. (A) After recording of the 1st response (GABA(1st) in A, C), 1mM GABA was applied to the same cells in the presence of 100 µM PTX (GABA(2nd) in A). PTX reduced the second GABA currents to 10 ± 2 % of the 1st ones (n = 6 cells). **: $p < 0.01$; paired t-test. (B, C). Repeated GABA application had no marked influences on the second responses. The amplitude of 2nd GABA currents were diminished to 84 ± 3 % of the 1st ones (n = 7 cells) ($p = 0.5$ by paired t-test) (B, C).

Suppl. Fig. S2

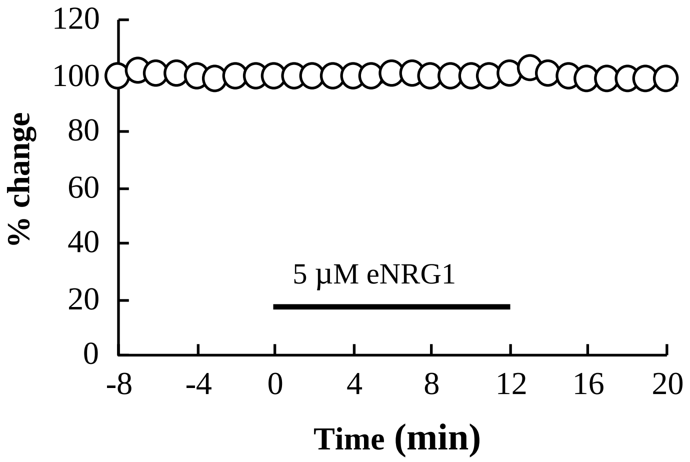

Suppl. Fig. S2
Acute eNRG1 application had no marked influence on single unit activity of nigral dopamine neurons. Single unit recording was performed recorded at 32 °C from nigral dopaminergic neurons in midbrain slices. Mean basal firing rates for 8 min (2.4 ± 0.3 Hz) were set at 100 % and compared with the mean frequency at 11-12min in the presence of 5 µM eNRG1 (n = 6, p = 0.9 (paired t-test)). Horizontal slices were prepared from juvenile mice at postnatal 3 week.
